# Notes and News

**Published:** 1900-09-22

**Authors:** 


### Notes and News.

A bed in the Edinburgh Royal Infirmary and one in the St. Andrews Cottage Hospital is to be endowed through the contributions made to perpetuate the memory of Lieutenant Tait, of the Black Watch, the celebrated golf champion, who lost his life in South Africa.

A decree has been passed in Austria permitting women to obtain medical degrees at the Austrian Universities after having completed the necessary studies. Up to the present time women have been allowed to go through the whole medical curriculum, but not to graduate.

An extraordinarily severe epidemic, in some way connected with the great heat, has been prevalent in Hamilton, Ontario. The form of the attack is of the nature of dysentery. Whole factories have had to suspend work, and hotels and other public establishments have suffered.

The report of Dr. Harris, medical officer to the Vestry of Islington, shows that more adulteration of milk takes place on Sunday than any other day in the week. The reason, probably, is that the demand for milk is greater on Sunday, and the easiest way to meet the extra demand is obviously to dilute the usual supply. Invalids and infants naturally suffer in consequence.

The first hospital, we believe, designed by a woman is to be erected at Mediasch, a town in Hungary. The architect, Erica Paulus, is said to be a young Swiss lady. In 1892 she obtained a post in the Town Engineer Bureau at Bistritz, and afterwards took up the study of architecture, and has designed some very successful buildings since.

Dr. Low and Dr. Sambon, who are doing experimental work in the Roman Compagna, have so far proved the possibility of taking precautions against malaria. Although the people around them have suffered severely with fever, they themselves have kept in excellent health. They are making interesting observations in the life history of mosquitos and other blood-sucking insects, and they have collected some valuable material for the benefit of the School of Tropical Diseases, of which Dr. Low is Craggs scholar.

Dr. S. Knopf, of New York, obtained the prize of 4,000 marks offered by the Berlin Congress on Tuberculosis for the best popular work on Tuberculosis as a Social Scourge, and the best means of prevention.

A recent inspection in Manila revealed the unpleasant fact that one hundred lepers were found concealed amongst the population. It is stated that there are about thirty thousand lepers in the Philippines.

New York will have to take some means similar to our own to stamp out rabies if the cases continue to be as numerous as during recent years. Seventeen deaths are reported from rabies during the past five years.

Great disappointment was felt at Glasgow when, after a cessation of new plague cases for some days, a further outbreak occurred. The total number of cases up to the present time have been 21. The persons now under observation number 110.

The fever report from Senegal is most alarming. St. Luis appears to have suffered most, and the condition of the inhabitants is reported as most distressing. Europeans making arrangements to leave found no transport available for some time, and in some parts not a single white man remained, all having fled from the spot who had not already become victims of yellow fever.

The City of Rochester Hospital in the United States has had the refusal of a legacy of 50,000 dollars by the will of the late Judge F. O. Mason, of Geneva, U.S.A. The condition attached to the legacy is that no chaplain shall be employed in the hospital, nor anyone else undertake the duties usually performed by the chaplain. If the managers do not agree, the money is to revert to the estate of the testator, who also decrees that not more than 10,000 dollars of the whole amount shall be expended on buildings

A section of the population of Aberdeen are still keenly working to secure the municipalisation of the Royal Infirmary. Of course, the question will arise as to whether it can any longer retain the title of "Royal" when differently constituted, as it will simply then be a rate-supported institution compulsorily maintained by the population, and not a monument to the generosity and philanthrophy of those who voluntarily supported it in the past, and support it in the present. The citizens who have the change in view, having formed a committee, decide that the present system of management is unsatisfactory, and propose that it should be placed in the hands of some popularly-elected board with statutory rating powers at its disposal.

"An Occasional Correspondent" writes from St. Moritz: "Learning that the President of the German Committee for the Investigation of Cancer (Professor von Leyden) was staying at Pontresina I called on him, and asked if the committee were accepting subscriptions towards the expenses of the investigation. He replied that the German Imperial and Prussian Governments were paying the expenses, and that, therefore, they did not require any pecuniary assistance; but that he would be very glad to receive any co-operation from any British society or individual investigator who is working at this subject, and who might be able to assist the Berlin committee in its work." Our correspondent asks us to bring this under the notice of our readers.

